# World Antimalarial Resistance Network I: Clinical efficacy of antimalarial drugs

**DOI:** 10.1186/1475-2875-6-119

**Published:** 2007-09-06

**Authors:** Ric N Price, Grant Dorsey, Elizabeth A Ashley, Karen I Barnes, J Kevin Baird, Umberto d'Alessandro, Philippe J Guerin, Miriam K Laufer, Inbarani Naidoo, François Nosten, Piero Olliaro, Christopher V Plowe, Pascal Ringwald, Carol H Sibley, Kasia Stepniewska, Nicholas J White

**Affiliations:** 1 International Health Program, Menzies School of Health Research and Charles Darwin University, Darwin, Northern Territory, Australia; 2 Centre for Vaccinology & Tropical Medicine, Nuffield Department of Clinical Medicine, Churchill Hospital, Oxford, UK; 3 Department of Medicine, University of California, San Francisco, CA, USA; 4 Epicentre, 8 rue Saint Sabin, 75011 Paris, France; 5 Division of Clinical Pharmacology, Department of Medicine, University of Cape Town, Cape Town, South Africa; 6 ALERTAsia Foundation, Eijkman Institute for Molecular Biology, Jakarta Pusat 10430, Indonesia; 7 Department of Parasitology, Institute of Tropical Medicine, Antwerp, Belgium; 8 Center for Vaccine Development, University of Maryland School of Medicine, Baltimore, MD, USA; 9 Malaria Research Programme, Medical Research Council, Durban, KwaZulu-Natal, South Africa; 10 Shoklo Malaria Research Unit, Mae Sot, Thailand; 11 UNICEF/UNDP/World Bank/WHO Special Programme on Research &Training in Tropical Diseases (TDR) World Health Organization, 20 avenue Appia, CH-1211, Geneva Switzerland; 12 Global Malaria Programme (GMP), World health Organization, 20 avenue Appia, CH-1211, Geneva Switzerland; 13 Faculty of Tropical Medicine, Mahidol University, 420/6 Rajvithi Road, Bangkok 10400, Thailand

## Abstract

The proliferation of antimalarial drug trials in the last ten years provides the opportunity to launch a concerted global surveillance effort to monitor antimalarial drug efficacy. The diversity of clinical study designs and analytical methods undermines the current ability to achieve this. The proposed World Antimalarial Resistance Network (WARN) aims to establish a comprehensive clinical database from which standardised estimates of antimalarial efficacy can be derived and monitored over time from diverse geographical and endemic regions. The emphasis of this initiative is on five key variables which define the therapeutic response. Ensuring that these data are collected at the individual patient level in a consistent format will facilitate better data management and analytical practices, and ensure that clinical data can be readily collated and made amenable for pooled analyses. Such an approach, if widely adopted will permit accurate and timely recognition of trends in drug efficacy. This will guide not only appropriate interventions to deal with established multidrug resistant strains of malaria, but also facilitate prompt action when new strains of drug resistant plasmodia first emerge. A comprehensive global database incorporating the key determinants of the clinical response with *in vitro*, molecular and pharmacokinetic parameters will bring together relevant data on host, drug and parasite factors that are fundamental contributors to treatment efficacy. This resource will help guide rational drug policies that optimize antimalarial drug use, in the hope that the emergence and spread of resistance to new drugs can be, if not prevented, at least delayed.

## Background

The emergence and spread of antimalarial drug resistance is one of the most important factors undermining malaria control programmes in most of the malaria endemic world. [1]. Following World Health Organization (WHO) recommendations, more than 60 countries have now adopted artemisinin-based combination therapies (ACT). Changing national policy in resource-poor settings has huge financial and practical implications and the debate continues as to which are the most suitable combinations and how these new treatments should be deployed and funded. In this context, it becomes of paramount importance to policy makers, funding bodies and researchers alike, to document the clinical efficacy of older drugs now being combined with artemisinins and to monitor the continued efficacy of newly deployed antimalarial regimens.

The number of clinical trials being conducted has risen dramatically over the last decade. This is primarily in response to an increasing appreciation of the major impact that antimalarial drug resistance has had on the health of tropical communities, the perceived need for the rational deployment of new regimens based on empirical evidence and a significant increase in the funding for these critical endeavours. Numerous groups have now generated a wealth of knowledge on different treatment regimens in different sites over a prolonged period of time. Unfortunately many studies remain unpublished. While each trial may have been designed with an immediate applicability for local policy makers or to test a specific hypothesis, an opportunity exists to extract information of global relevance.

**Figure 1:**
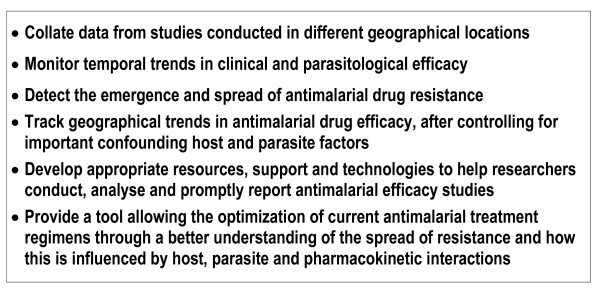
The broad aims of the global efficacy database.

Combining published and unpublished data from different sites into a comprehensive global database will provide the ability to monitor geographical and temporal trends in clinical efficacy to drugs that are being used as well as new drugs that are being evaluated. Such information will facilitate the early recognition of problems with antimalarial efficacy as they first emerge in affected areas and, perhaps more importantly, before they appear in adjacent areas. Prompt recognition of the earliest signs of compromised drug efficacy is particularly important now, as highly effective ACTs are increasingly becoming the first-line treatment in most of the world. A global database for monitoring clinical efficacy of antimalarial drugs linked to similar databases tracking *in vitro*, molecular and pharmacokinetic information will directly inform decisions about which drugs should be used in specific settings to assure adequate cure rates for afflicted populations (Figure 1). Equally important, this network will provide a comprehensive view of the evolution of resistance that may lead to new strategies for deploying effective antimalarial combinations in ways that will deter the emergence and spread of resistance.

## Rationale for an individual patient level database

**Figure 2:**
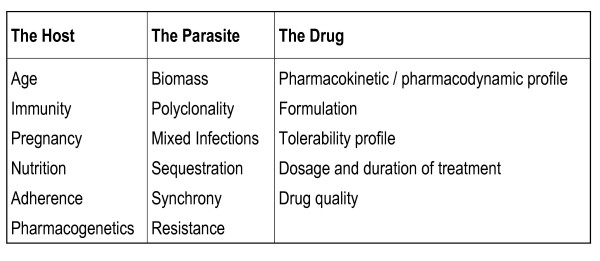
Determinants of *in vivo *response to antimalarial therapy.

The antimalarial drug response can be characterized through a triad of *in vivo*, *in vitro *and molecular studies. For clinicians and policy-makers the most informative measure on which to base treatment practices is the *in vivo *response of patients within a target population. However, the therapeutic response must be interpreted as an interaction between the host, the parasite and the administered drug (Figure 2). The cost and logistics of *in vivo *clinical studies severely limit the availability of these data. *In vitro *and molecular data provide additional information on parasite susceptibility in the absence of the confounding environment of the host, but often their clinical relevance is difficult to gauge.

Between 1966 and 2002, more than 435 antimalarial drug trials involving over 80,000 patients were published [2]. There is also a large number of studies [3]. Furthermore, there is frequently a delay of several years between the completion of a study and its publication, often related to difficulty in presenting and analysing the data. In order to leverage this huge and evolving resource to provide stakeholders with the current status of antimalarial efficacy, the available data must be systematically gathered, collated, analysed and presented in a timely fashion. The current heterogeneity in analyzing and reporting such studies is a major impediment to reaching this goal.

Systematic review provides one approach to combining data but it lacks the power of large individual patient analyses and is not amenable to standardizing analytical methods or sub-group analyses. Meta-analyses of published studies pool data from multiple studies, but these studies tend to focus on specific treatment regimens and differences between treatments in comparative studies rather than providing standardized estimates of drug efficacy across different studies. Aggregating data from published reports can provide useful indicators of the global situation of antimalarial resistance [3]. However in the absence of individual patient level data, this approach is limited by the diversity of study designs and analyses and variations in the populations being studied.

An alternative approach is to gather individual patient data from published and unpublished studies [4]. Although protocols for conducting antimalarial efficacy trials have been standardized to some extent by the WHO, the recommendations have been revised three times over the last decade, thus limiting comparisons of historical data. For example, the duration of follow-up is a key factor that determines the derived estimates of drug efficacy. Until recently, WHO recommended a 14-day period of follow-up in areas of high transmission, although this short follow-up period was also often used in intermediate and low transmission settings. When resistant isolates first emerge in the parasite population, parasite recrudescence tends to occur late (up to 63 days after the initial treatment for drugs with long terminal elimination half-lives). Studies with less than 28 days follow-up, therefore, do not adequately assess the true cure rate and are unable to detect early evidence of resistance [5]. By the time that early treatment failures occur and recurrent infections are observed before day 14, parasite resistance is already firmly established [6]. Further confusion arises from the distinction between the definitions of late clinical and parasitological failure. Previous WHO protocols considered treatment failure in high transmission areas only if recurrent parasitaemia was accompanied by fever. In low transmission settings, where most people with recurrent parasitaemia become symptomatic, studies generally define failure as occurrence of any parasitaemia, irrespective of symptoms. These fundamental differences in defining the main study endpoints undermine comparison of drug efficacy between low and high transmission sites and severely compromise analysis of geographic trends in antimalarial drug resistance. In 2001 the WHO protocol was revised again to follow patients for at least 28 days [7], defining failure as the recurrence of any recrudescent parasitaemia, even without symptoms. Although such changes will help standardize estimates of efficacy, the longer follow-up requires that the studies should be complemented by molecular genotyping to distinguish recrudescence (true treatment failure) from reinfection. Currently there are no agreed guidelines on how this critical distinction should be made [8].

Despite these improvements in the most recent WHO protocol, variations in study design by individual investigators are likely to continue. In addition, differences in study populations with respect to key determinants of antimalarial response, such as patient age and parasite density [9], will continue to make comparison of estimates of treatment efficacy across different studies challenging.

Even with standardized study design and enrollment criteria, derived estimates of efficacy presented in comparative drug trials can vary significantly as a result of differences in the statistical analysis [10]. To maintain a conservative analytical approach, comparative drug studies are often constrained to per-protocol populations or emphasis is placed on intention-to-treat analyses. Although these methods maintain an internal validity, they introduce important biases in the analysis when combining patients who were followed for different periods. The duration of follow-up varies due to differing study designs, patient adherence, and reinfection and relapse rates, and together these variables can change the evaluable population in the study by up to 50%. Efficacy estimates have fluctuated by more than 10% when the same study is evaluated by these different protocols [11]. Since the latest WHO guidelines state that a treatment regimen should not be considered effective if the risk of recrudescence exceeds 10%, variations in efficacy estimates entirely attributable to statistical methodology can have important implications on policy decisions.

A primary goal of a comprehensive global data base is to allow antimalarial drug efficacy to be monitored and compared between diverse sites and over time. To achieve this, the clinical and parasitological data need to be analysed at an individual patient level. This approach allows the attributable component of parasite resistance to be established after minimizing the confounding of host and drug-related factors. The additional advantage of this approach is that when available in vitro, molecular and pharmacokinetic data exist they can be linked at an individual level, leading to a better understanding of how these variables correlate with the therapeutic response.

## The Challenge

There are two main challenges that must be addressed before standardized efficacy estimates can be derived from a global database. First, a simple, reliable and comprehensive method to organize a core set of primary data from different study sites and research groups must be established. Second, an appropriate *a priori *analytical approach is needed that yields core outcome measures minimizing potential biases and that accommodates most of the inevitable variation in study design, recruitment and follow-up.

**Figure 3:**
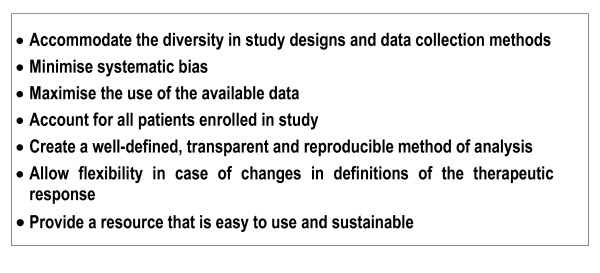
The major goals of the analytical and methodological approach to collating data.

The primary goal of a global database is to generate comparable and reliable estimates of antimalarial drug efficacy, across a wide range of studies from different geographical areas. The aim is to produce and disseminate standardized estimates of efficacy that will be readily accessible to researchers and policy makers alike (Figure 3). The initial focus will be to generate overall measures of efficacy stratified by key confounding factors such as patient age and parasitaemia. More complex correlations with in vitro, pharmacokinetic and molecular markers of drug resistance could be explored later.

## The Solution

All efficacy studies begin with patients enrolled in a study protocol and treated for malaria. Most studies end when the patient meets the criteria for failure or the patient is followed for some pre-determined duration without meeting criteria for failure. Given variations in duration of follow-up, survival analysis is generally regarded as the most appropriate analytical approach [12,7,10]. If a patient observation period ends prematurely and treatment failure did not occur, then that patient is "censored" at the time s/he was last observed.

The use of survival analysis offers the following important advantages :

1) Data from patients with different follow-up periods can be combined and efficacy estimates generated at different time points. These can then be compared between studies with different length of follow-up.

2) All available data contributes in the analysis, thus increasing the precision of the derived estimates.

3) The approach avoids systematic biases introduced by dropping patients from the analysis that do not complete follow-up (per-protocol analysis) or classifying patients as failures who do not represent true biological failures (intention-to-treat analysis).

In survival data each patient is characterized by a period of observation and whether or not treatment failure occurred during this observation period (the "status"). This can be derived from four key parameters:

1) The last day of follow-up

2) The patient outcome on the last day of follow-up

3) The parasite species on the day of failure

4) The genotyping results (recrudescence, reinfection, no result)

The outcomes for all patients on the last day of follow-up need to be categorized into mutually exclusive groups that cover all possible endpoints (Table 1). These can be broadly divided into three groups.

**Table 1:** Key variables requested by the global efficacy database

Time and space variables#	Baseline variables	Primary outcome variables	Secondary outcome variables *
Study Site Study identifier Date of enrollment	Unique patient ID Treatment Parasite species Age Parasite density Patient weight	Last day of follow-up Outcome on last day of follow-up Species on day of failure Genotyping result on day of failure	Parasite clearance Fever clearance Change in haemoglobin Gametocyte carriage Adverse events

Variables in bold represent the key parameters necessary for deriving estimates of efficacy for each regimen.

* Additional variables that could be incorporated for analyses of secondary outcomes

# Study site details will be collated separately from the data at the individual patient level., and filed once for any study but linked to each individual patient record.

For more details see [13]

1) Patients who complete the study and do not meet criteria for treatment failure. For these patients, the last day of follow-up is the full duration of the study period.

2) Patients who meet criteria for treatment failure; in this case the last day of follow-up is the day when treatment failure occurred.

3) Patients who neither complete the full follow-up period nor meet the criteria for treatment failure (e.g. lost to follow-up, withdrawal of consent, use of other antimalarials, protocol violations, infection with other species etc.). For these patients, the last day of follow is the day when the patient was last observed.

Since the primary objective is to determine the prevalence of parasite resistance, failure is defined as early treatment failure during the first few days after the start of treatment or the first reappearance or persistence of parasitaemia during subsequent follow-up. These definitions are consistent with the current WHO guidelines.

Condensing the patients' *in vivo *response into the four simple parameters listed above, allows one to generate in a transparent and flexible manner, the status of the patient required for survival analysis. Table 2 shows an example of a coding table which allows reviewers to appreciate how various protocol violations were dealt with and thus the shortcomings of the derived estimates. If additional baseline data are available, one can then stratify the efficacy estimates according to important baseline line confounding variables (Table 3).

**Table 2:** Description of possible outcomes on the last day of follow-up for patients enrolled in clinical efficacy studies

Variable code	Description
	Patients who complete the study

0	ACPR
1	ETF and Death
2	ETF with Severe Malaria
3	ETF with Danger Signs
4	ETF with Parasitological Criteria (day 2 > day 0 or day 3 > 25% day 0)
5	ETF with Clinical Criteria (documented fever and parasitaemia on day 3)
6	ETF not otherwise specified (for when details of why ETF classified not available)
7	LCF and Death
8	LCF with Severe Signs
9	LCF with Danger Signs
10	LCF with fever (either measured or subjective)
11	LPF
12	LPF/LCF Indistinguishable (for when details of why LCF/LPF classified not available)
	Patients who do not complete the study
13	Adverse event requiring change in therapy prior to completion of full dose
14	Protocol violation
15	Death not due to malaria
16	Lost to follow-up
17	Use of other antimalarials outside of study protocol in the absence of parasitaemia
18	Withdrawal of consent by patient prohibiting further follow-up
19	Investigator initiated withdrawal from further follow-up
20	Patient who does not complete follow-up for any other reason not listed above
21	Enrolment Violations

ACPR – Adequate Clinical and Parasitological Response; ETF – Early Treatment

Failure; LPF – Late Parasitological Failure; LCF – Late Clinical Failure

**Table 3:** The derivation of the patients status on the last day of follow, which is required for survival analysis

Key Variable				Status		
Species at Enrollment *	Outcome †	Species at Failure *	PCR Correction **	PF Unadjusted by genotyping φ	PF Recrudescence φ	PV Recurrence φ

1–5	0	N/A	N/A	0	0	0
Any	1–6	1	N/A	1	1	0
Any	1–6	2	N/A	0	0	1
1 or 3 or 4	7–12	1 or 4	1	1	0	0
1 or 3 or 4	7–12	1 or 4	2	1	1	0
1 or 3 or 4	7–12	1 or 4	3	1	§	0
1 or 3 or 4	7–12	3	1	1	0	1
1 or 3 or 4	7–12	3	2	1	1	1
1 or 3 or 4	7–12	3	3	1	§	1
1 or 3 or 4	7–12	2 or 5	N/A	0	0	1
1 or 3 or 4	7–12	6	N/A	0	0	0
2 or 5	7–12	1 or 4	N/A	1	-	0
2 or 5	7–12	3	N/A	1	-	1
2 or 5	7–12	2 or 5	N/A	0	-	1
2 or 5	7–12	6	N/A	0	-	0
1–5	13–20	N/A	N/A	0	0	0
1–5	21	N/A	N/A	-	-	-

* 1 = Pf monoinfection; 2 = Pv monoinfection; 3 = Pf + Pv mixed; 4 = Pf mixed, but Pv not present; 5 = Pv mixed, but Pf not present, 6 = Other Species not Pf or Pv

** 1 = Reinfection, 2 = Recrudescence, 3 = Unavailable/Uninterpretable; N/A = Not applicable

† See Table 2 φ 0 = Not a failure; 1 = Failure

§As yet there is no consensus as to how to deal with indeterminate PCR results, although strategies to deal with these are being developed. Currently these are excluded from the PCR corrected analysis.

The proposed system standardizes the process of data collection in a robust and yet flexible manner, allowing an analysis that accommodates the inevitable diversity of study methodologies and collection methods. Importantly it still retains the ability to present alternative analyses, such as proportions of failures on any particular day. Whatever analytical method is used the process should be transparent to analysts interpreting the findings. Such transparency is now evident from the coding table (Table 2). This approach of using standardized terminology greatly facilitates the ability to upload and cross check individual patient study data to a global resource. Once pooled estimates of efficacy can be readily generated and compared across geographically and temporally disparate studies.

Data can be gathered retrospectively from completed studies but this may require recoding of the data to produce the key parameters outlined in Table 3 (for further details see reference [13]. If the collection of these key parameters are incorporated prospectively into studies, then the process is simplified greatly.

## The Outputs

The global database will initially focus on deriving six standardized efficacy estimates: the cumulative incidence of *Plasmodium falciparum *recurrence (unadjusted and adjusted by genotyping) up to day 28 and day 42 and the cumulative risk of recurrence with *Plasmodium vivax *at day 28 and day 42. Subgroup analyses could be performed selecting patients according to certain age and parasitaemic limits to minimize the confounding of immunity and parasite biomass.

Although the risk of treatment failure is the main component of the therapeutic response, other parameters of interest can also be included, such as parasite and fever clearance times, haematological recovery, gametocyte carriage, and drug safety and tolerability (Table 3). As data collection methods become standardized (see below), it will become feasible to link and present these data.

To demonstrate the utility of such a system data have been collected from 82 studies with 162 treatment arms from 25 countries. In total individual records from 25,214 patients have already been pooled and the day 28 recurrence rates estimated (PCR adjusted and unadjusted). The latter are available online [13] along with an example of a single study contribution and details on how the data should be coded. It is important to emphasize that if data are collected in this simplified mode at the outset, then the cumulative incidence of failure for each study can be generated easily using survival analysis, whilst retaining the ability to peruse other statistical outputs.

## Potential for future developments

The goal of a global database is to characterize temporal and geographical trends in antimalarial efficacy, allowing information-based decision making among health policy developers and implementers. Retrospective analysis of the demise of old drugs could potentially provide useful information on how a similar fate might be avoided when deploying new treatments. Ultimately, the strength of an international resource lies in its ability to collate and report rapidly the current data on antimalarial efficacy. This will ensure that the international community has the best opportunity to detect and react to the earliest warnings of failing drug regimens. The additional benefit of documenting what is currently known is that it becomes apparent where important gaps in the data exist, thus helping to prioritize clinical and parasitological studies of antimalarial efficacy in these areas.

Another important component of the World Antimalarial Resistance Network will be the development of additional resources to increase local capacity to conduct and interpret clinical trials. In this respect an open-access software program will be developed to provide a universal database system ready for sites to implement. This free software would provide a much needed service to clinical investigators conducting antimalarial efficacy trials in malaria endemic countries. At the same time, the data would be collected in a format that could easily be uploaded to the global database, at the discretion of the local study team. The system would also promote adherence to standardized protocols and facilitate the collection of the most critical variables for basic efficacy and safety measurements.

As the data from the other global databases proposed in this edition of the journal accrue, the opportunity will arise to cross-link molecular, *in vitro *susceptibility assays and pharmacokinetic parameters with the clinical data. This can be done both at the population (in time and place) or individual level. Such merging will allow the investigation and validation of surrogate markers of clinical efficacy. The power of a comprehensive data collection would allow a multivariate analysis to identify parameters, other than parasite resistance, associated with the therapeutic outcomes. This information could then be used to rationalize public health strategies (e.g. optimizing dosing recommendations and identification of groups of patients at particular risk).

## Challenges ahead

There are numerous challenges to interpret the data that have already been gathered, and to develop acceptable and reliable methodologies that will improve study design and data collection and analysis in the future. The first step toward these goals is to ensure that research groups, policymakers, governing bodies and funding agencies perceive tangible benefits that can be accrued from sharing both data and methodologies. These benefits need to be balanced by the perceived risks to ownership and privacy when releasing individual patient data to a third party. Transparent protocols need to be developed that comply with international ethical standards, protect intellectual property rights, and ensure that patient data remain confidential.

Five key variables are required to derive efficacy estimates for each treatment (Table 3). Subgroup analyses by and large are fairly standard and amenable to strict definitions. The major exception to this is the genotype of the recurrent infection (recrudescent, reinfection or unavailable/uninterpretable). The methodology used to derive these three categories varies markedly between research units, with some using one marker and others a multitude of markers [8]. In areas of high transmission, the interpretation of these parameters in polyclonal infections is at best challenging and at worst impossible. Debate on how to deal with these issues is gathering momentum and in the coming year, it is hoped that consensus and recommendations for standard approaches will be agreed upon. In the meantime there is no option but to rely on local practices. However since the genotype parameter remains a crucial component for estimating true recrudescence, details of these methods and protocol design must accompany all contributions to the global database, so that the validity of the data can be assessed.

Where drug efficacy surveillance systems are being developed the benefits of adopting established methodologies and analytical approaches are compelling. However it is often difficult to change a system that is already in place and appears adapted to local needs. The challenge is to ensure that the proposed changes in the coding of data are simple and easily incorporated into established practices. Importantly the uniform collection of the key variables advocated in this paper will circumvent the inevitable variations in protocol design and execution, which reflect local requirements and capacities. From the feedback of the participants in a pilot project, it appears that this is so, and that, importantly, the process of cleaning, validating and analyzing data is greatly improved. This is currently an evolving system and, therefore, there are likely to be problems that need to be resolved and areas that need further clarification. Feedback from those involved in conducting efficacy studies is welcomed and can be registered as a readers comment on this article.

## Conclusion

In the last ten years the emergence of multidrug resistant strains of plasmodia raised the spectre of untreatable malaria for poorly resourced communities. The development of ACTs has brought the hope of not only rescuing some compromised antimalarial drugs, but also delaying the emergence and spread of new resistant strains. In addition improved funding has paved the way for the discovery of novel antimalarial compounds, some of which should become available in the next two to ten years. Although the next ten years look much brighter for malaria treatment programmes, it would be a disaster if that led to complacency. Plasmodia have proved efficient at evolving resistance to novel agents and this is likely to continue. Hence it is vital that prospective monitoring of antimalarial drug efficacy remains an international priority, with estimates of clinical efficacy and *in vitro *susceptibility testing central to detecting early warning signals of resistance to ACTs. Furthermore developing a global resistance database to track clinical efficacy will help rationalize treatment practices, ensure ongoing surveillance, and identify factors most conducive to maintaining parasite susceptibility to available treatment regimens.

## Competing interests

The author(s) declare that they have no competing interests.
